# Elevated IL-33 promotes expression of MMP2 and MMP9 via activating STAT3 in alveolar macrophages during LPS-induced acute lung injury

**DOI:** 10.1186/s11658-018-0117-x

**Published:** 2018-10-31

**Authors:** Yafeng Liang, Nengli Yang, Guoquan Pan, Bingxin Jin, Shufen Wang, Wei Ji

**Affiliations:** 1 Department of Pediatric Pulmonology, Children’s Hospital of Soochow University, No 303, Jingde Road, Suzhou, 215003 China; 2 Department of Pediatric Intensive Care Unit, The Second Affiliated Hospital & Yuying Children’s Hospital, Wenzhou Medical University, Wenzhou, 325000 China; 3 Department of Anesthesiology, The First Affiliated Hospital of Wenzhou Medical University, Wenzhou, 325000 China

**Keywords:** Acute lung injury (ALI), IL-33, MMP2, MMP9, STAT3

## Abstract

**Background:**

Pulmonary inflammation and endothelial barrier permeability increase in acute lung injury (ALI) and acute respiratory distress syndrome (ARDS) induced by pro-inflammatory cytokines and matrix metalloproteinases (MMPs). However, the relationship between pro-inflammatory cytokines and MMPs in ALI/ARDS remains poorly understood.

**Methods:**

A lipopolysaccharide (LPS)-induced ALI rat model was established through intratracheal instillation. The wet/dry ratios of lung tissues were measured, and bronchoalveolar lavage fluid (BALF) was collected to test protein concentrations, total cell/macrophage numbers, and pro-inflammatory cytokine levels. LPS-treated alveolar macrophages were utilized in in vitro experiments. The expression and secretion of MMPs were respectively detected using quantitative PCR, Western blotting and ELISA assays.

**Results:**

The levels of IL-33 and MMP2/9 in BALF increased in all the ALI rats with severe lung injury. LPS-induced IL-33 autocrine upregulated the expression of MMP2 and MMP9 through activating STAT3. Neutralizing IL-33 in culture medium with specific antibodies suppressed the expression and secretion of MMP2 and MMP9 in LPS-treated alveolar macrophages. Consistently, eliminating IL-33 decreased the levels of MMP2 and MMP9 in BALF and alleviated lung injury in ALI rats.

**Conclusion:**

The IL-33/STAT3/MMP2/9 regulatory pathway is activated in alveolar macrophages during acute lung injury, which may exacerbate the pulmonary inflammation.

## Background

Acute lung injury(ALI), also known as acute respiratory distress syndrome (ARDS), is characterized by a serious inflammatory reaction in the lung, and leads to serious hypoxaemia and poor pulmonary compliance in both children and adults [1, 2]. Disruption of the alveolar–capillary unit, breakdown of the barrier, and gas exchange are clinical manifestations of ALI/ARDS [3]. Although new therapies, such as extracorporeal membrane oxygenation and lung protective ventilation strategy, have been applied, the mortality rate of ALI patients is still as high as 20–50% [4]. Thus, it is urgent to develop more effective treatments for ALI.

The uncontrolled inflammatory response is a key step during the development of ALI/ARDS. Pro-inflammatory immune cells and the cytokines released by them can increase the permeability of the alveolar–capillary barrier, leading to pulmonary dysfunction [5]. Alveolar macrophages (AMs) are immune cells accounting for 95% of airspace leukocytes [6]. An essential process in ALI, activation of AMs leads to the oversecretion of various inflammatory cytokines, such as TNF-α, IFN-γ, and interleukins [7]. As a newly identified member of the interleukin (IL)-1 family, IL-33 can aggravate inflammatory reactions and increase microvascular permeability, two processes that decrease the survival rate in LPS-induced ALI mice [8]. In our previous study, the plasma level of IL-33 was found to be higher in ALI patients than in the healthy [9]. This evidence suggests a potential association between IL-33 and ALI.

Matrix metalloproteinases (MMPs) are involved in ALI. MMPs-mediated degradation of proteins in the alveolar epithelial–endothelial unit, including intercellular junction proteins, basement membrane (BM) and proteins anchoring cells to the BM, is a central process in ALI [10]. Among these MMPs, MMP2 and MMP9 are being intensely studied, and their levels are elevated in BALF from ALI patients [11]. The in vivo lung injury model demonstrated a rise of both MMP-2 and -9 in BALF that correlated with the alveolar–arterial oxygen gradient (PA-a,O_2_) [12]. Also, in an immune complex deposition model, mice deficient in MMP-9 developed less severe lung injury than wild type mice [13]. However, the mechanisms underlying the rise of MMP2/9 during ALI remain elusive.

Here, we investigated the association between IL-33 and MMP2/9 in an LPS-induced ALI model in vitro and in vivo. Oversecretion of IL-33 promoted the expression of MMP2/9 by activating STAT3 in LPS-treated alveolar macrophages. The regulatory mechanism was further researched with LPS-induced ALI rats.

## Materials and methods

### LPS-induced ALI rat model

All 6-week old male Wistar rats were obtained from Wenzhou Medical University and maintained in a pathogen-free environment. Nine rats were assigned to each treatment group and a control group. The ALI model was established following the well-established protocol. In brief, the rats were anesthetized with 3% sodium pentobarbital, followed by the instillation of 2 mg/kg LPS (Sigma-Aldrich, St. Louis, MO, USA) solution into the tracheas. Instead of LPS solution, an equal volume of normal saline was used in the control group. For neutralizing IL-33, the rats received an intratracheal instillation of anti-IL-33 antibodies (5 μg, ab187060, Abcam, Cambridge, UK) or isotype IgG (ab172730, Abcam) 1 h after the LPS treatment. Then the rats were allowed to recover and were euthanized 24 h later with pentobarbital. All animal procedures were approved by the Ethics Committee of the Second Affiliated Hospital of Wenzhou Medical University.

### Lung wet/dry ratio analysis

After the rats were euthanized, their lungs were harvested and weighed immediately. Then the blood on the lung surface was washed away, and the lungs were dried at 60 °C for 72 h. The dried specimens were weighed again, and the wet/dry ratio was calculated accordingly.

### BALF collection and inflammatory cell analysis

The BALF was collected at 24 h after LPS treatment. Using a tracheal cannula, the lung was washed with 2 ml of normal saline three times. All flushing fluid was collected. The lavaged sample was then centrifuged at 1500×g for 10 min at 4 °C and the supernatant was collected for protein concentration analysis. Total protein concentration in the supernatant was measured. Each cell pellet was re-suspended in PBS and the total cell number determined in an automatic blood cell analyzer (Sysmex). Macrophages were marked with F4/80 antibodies or FITC-secondary antibodies and then analyzed and sorted with flow cytometry.

### Enzyme-linked immunosorbent assay (ELISA)

Concentrations of IL-33 (BMS2048), TNF-α (KRC3011), MMP2 (KHC3081), MMP9 (BMS2016–2), TIMP1 (ERTIMP1), IL-6 (BMS625), IL-10 (BMS629), and IFN-γ (BMS621) in BALF or cell culture medium were determined using their specific enzyme-linked immunosorbent assay (ELISA) kits (Thermo Fisher Scientific) according to the manufacturer’s instructions.

### Cell cultures and treatment

NR8383 AMs (Cell Bank of the Chinese Academy of Sciences, Shanghai, China) were derived from Sprague Dawley rats and cultured with Ham’s F-12 K medium containing 15% FBS (Gibco). 1 × 10^6^ NR8383 cells were stimulated with 1 μg/mL LPS (Sigma-Aldrich) or 100, 200, 400 pg/mL recombinant IL-33 (Novoprotein, Shanghai, China). For neutralizing IL-33, IL-33 antibody was added to the culture medium of NR8383 cells 1 h after the LPS treatment.

### Western blotting

The whole cell protein was obtained with cold cell lysis buffer and the total protein concentration was measured using the Bradford protein assay (Bio-Rad, Hercules, CA, USA). Equal amounts of protein were separated on 8–12% SDS-PAGE gel and transferred to a nitrocellulose membrane. The membrane was blocked with 5% milk and then incubated with primary antibodies (MMP2 (ab92536), MMP9 (ab38898), ST2 (ab228543), IL-1RAP (ab8110), and p65 (ab16502), Abcam, 1:1000; p-STAT3 (Y705, #4113), STAT3 (#12640), p-MAPK (#4511), and MAPK (#9212), Cell Signaling Technology, Beverly, MA, USA, 1:1000; H3 (sc-517576) and GAPDH(sc-32233), Santa Cruz, Dallas, TX, USA, 1:2000) at 4 °C overnight. Next, the membranes were incubated with appropriate secondary antibodies at room temperature for 1 h. IRDye 800CW- or IRDye 680-conjugated secondary antibodies (1:10000) were used for staining and then the proteins were detected using an Odyssey infrared imaging system (both from LI-COR, Lincoln, NE, USA).

### Quantitative real-time PCR

Extraction of total RNA from NR8383 cells was performed with RNAiso Plus reagent and further reverse-transcribed using a PrimeScript RT reagent kit (both from Takara, Tokyo, Japan). SYBR-Green mix (Roche) was used to carry out quantitative PCR according to the manufacturer’s instructions. Target gene expression was normalized to β-actin levels in respective samples as an internal control and calculated using the 2^−ΔΔCq^ method, and the relative mRNA expression was further calculated through normalizing to the control group.

### Statistical analysis

The software SPSS 13.0 and GraphPad Prism 5 were used in the statistical analyses. Group distributions were performed with Student’s t-test or analysis of variance (ANOVA). *P* < 0.05 was considered statistically significant.

## Results

### Establishment of acute lung injury (ALI) model

**Fig. 1:**
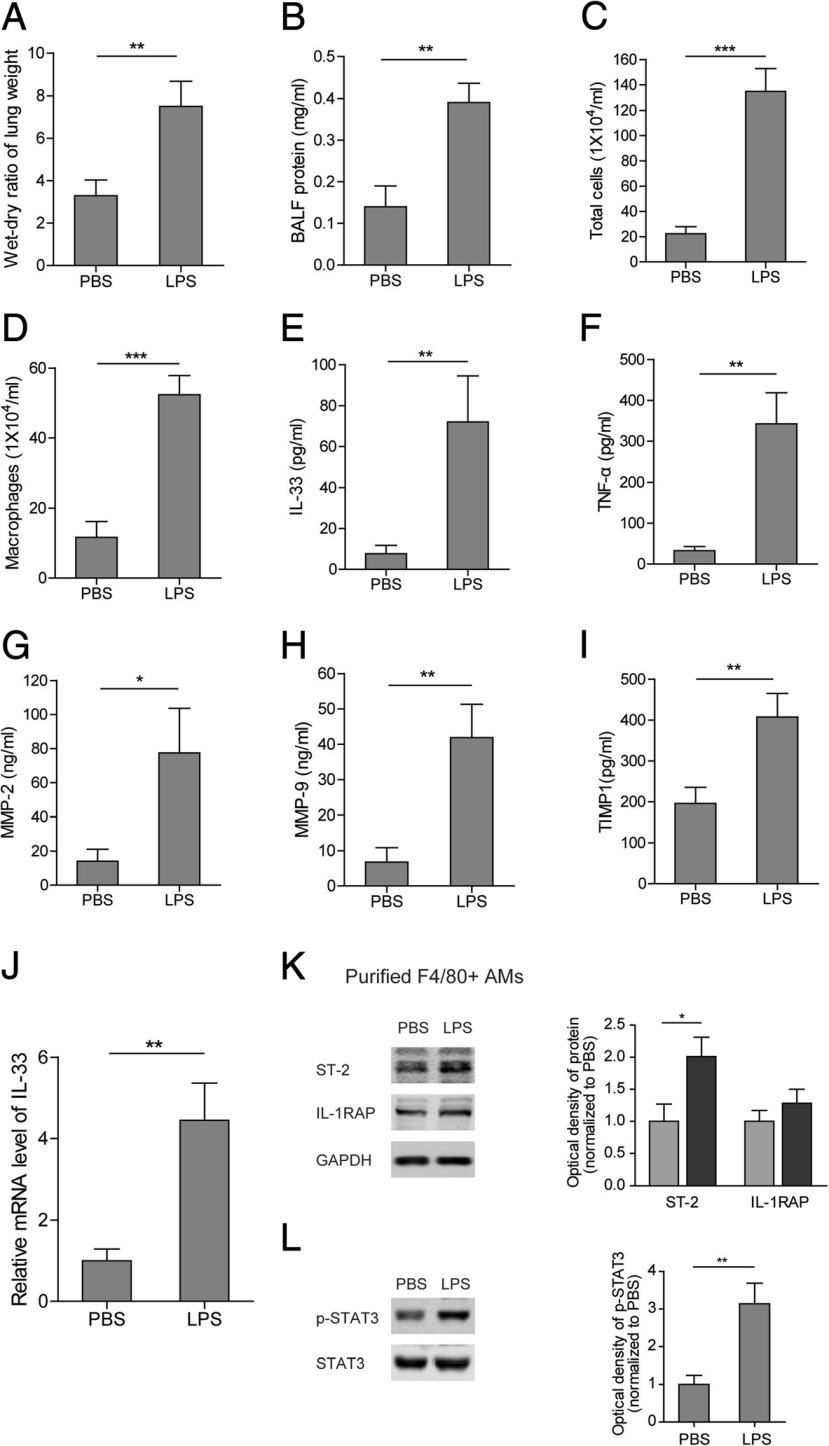
Establishment of LPS-induced ALI rat model. (**a**-**d**) LPS-induced lung injury in experimental rats, as characterized by lung wet/dry ratio (A), protein contents in BALF (**b**), total cells in BALF (**c**) and macrophages in BALF (**d**). (**e**-**i**) Levels of IL-33 (**e**), TNF-α (**f**), MMP2 (**g**), MMP9 (**h**), and TIMP1 (**i**) in BALF of LPS-induced ALI rats. (**j**) mRNA level of IL-33 in F4/80+ AMs purified from BALF of LPS-induced ALI rats. (**k**) Protein levels of ST-2 and IL-1RAP in F4/80+ AMs purified from BALF of LPS-induced ALI rats. (**l**) Protein levels of p-STAT3 and STAT3 in F4/80+ AMs purified from BALF of LPS-induced ALI rats. Data are shown as means±SD (*N* = 9 per group). Two-tailed *t* test was used in the statistical analyses. * *p* < 0.05, ** *p* < 0.01, *** *p* < 0.001 vs. control

In order to establish the in vivo ALI model, LPS was used to induce ALI in the rats. The lung tissues of ALI rats were collected at 24 h after treatment with or without LPS. The results showed that LPS caused severe pulmonary edema, as indicated by an increased lung wet/dry ratio (Fig. 1a). This observation was further confirmed by the increase of protein concentration in BALF from LPS-treated rats (Fig. 1b). To investigate the level of pro-inflammatory cells and cytokines in ALI, infiltration of total cells and macrophages was evaluated after LPS treatment. The total cells and macrophages were evidently enriched in BALF from LPS-treated rats (Fig. 1c and d). Additionally, the concentrations of IL-33, TNF-α, MMP2, MMP9 and TIMP1 in the BALF were significantly elevated (Fig. 1e-i). Furthermore, we purified the alveolar macrophages (AMs) with positive F4/80 staining from BALF. Consistently, the AMs from LPS-treated rats showed a higher level of IL-33 mRNA than those from the control group (Fig. 1j). Notably, as an IL-33 receptor, the ST-2 level was increased in the AMs from LPS-treated rats but the IL-1RAP level remained unchanged (Fig. 1k). Considering the inflammatory response induced by LPS, the status of the inflammatory signaling, p-STAT3, was also examined in the purified AMs. As shown in Fig. 1l, the level of p-STAT3 was significantly increased in the AMs purified from LPS-treated rats. Altogether, our findings indicated that the acute pulmonary inflammatory response was triggered in LPS-induced ALI rats.

### LPS induced expression of ST-2 and secretion of IL-33, TNF-α, MMP2, and MMP9 in primary AMs

**Fig. 2:**
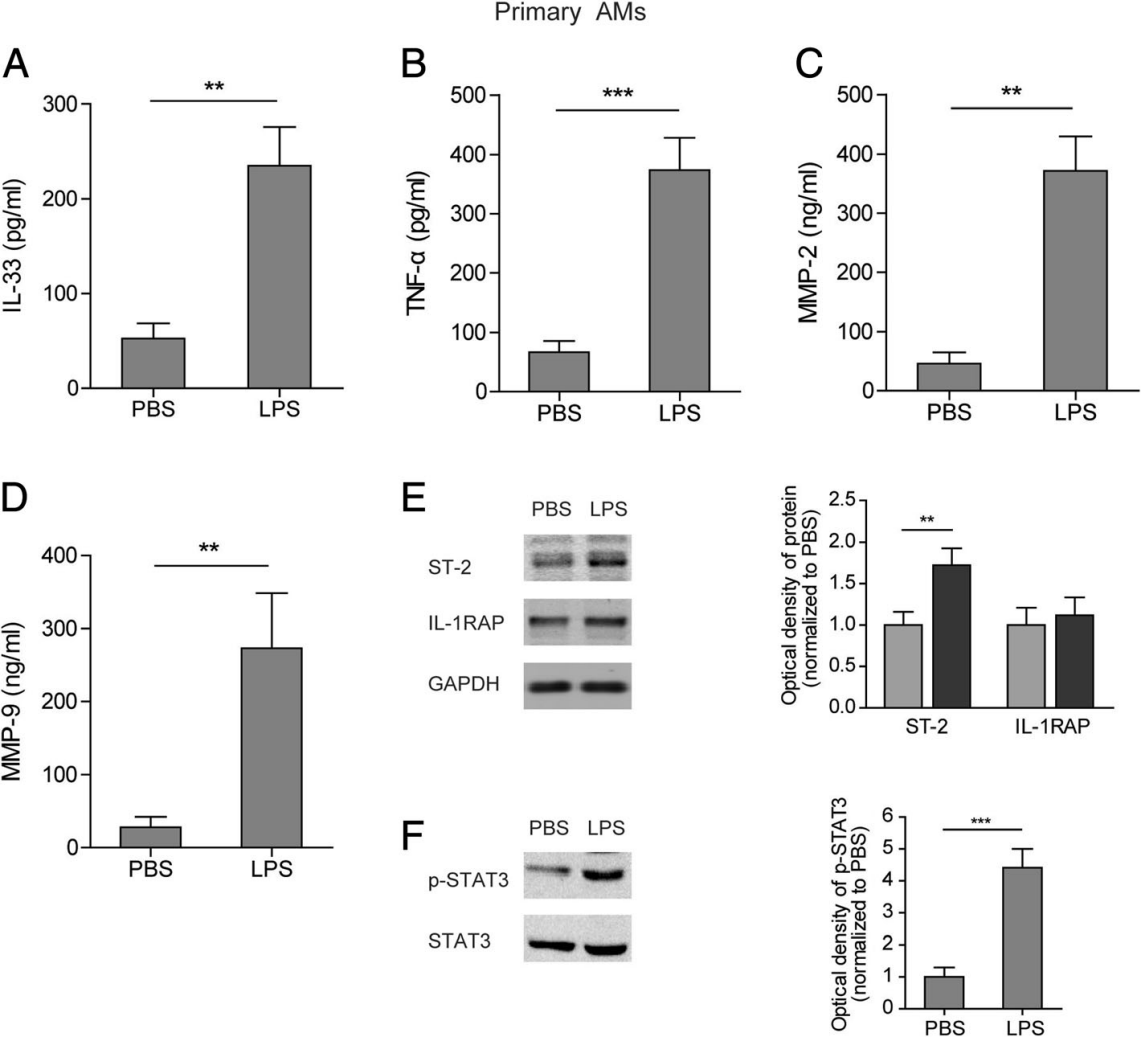
Levels of several inflammatory cytokines in culture medium of primary AMs treated with LPS. **(a**-**d)** Levels of IL-33 (**a**), TNF-α (**b**), MMP2 (**c**), and MMP9 (**d**) in the culture medium of primary AMs treated with or without LPS. (**e**) Protein levels of ST-2 and IL-1RAP in primary AMs treated with or without LPS. (**f**) Protein levels of p-STAT3 and STAT3 in primary AMs treated with or without LPS. Data are shown as means±SD (three independent repeats). Two-tailed *t* test was used in the statistical analyses. ** *p* < 0.01, *** *p* < 0.001 vs. control

Primary AMs were obtained through purifying the alveolar macrophages (AMs) in BALF from normal rats. LPS induced secretion of IL-33, TNF-α, MMP2, and MMP9 in primary AMs (Fig. 2a-d). Meanwhile, ST-2 was upregulated but IL-1RAP showed no change (Fig. 2e). STAT3 was also activated by LPS in the primary AMs (Fig. 2f). Therefore, the results suggest that primary AMs can be activated by LPS.

### LPS induced secretion of IL-33, TNF-α, MMP2, MMP9, and TIMP1 in AM cell line NR8383

**Fig. 3:**
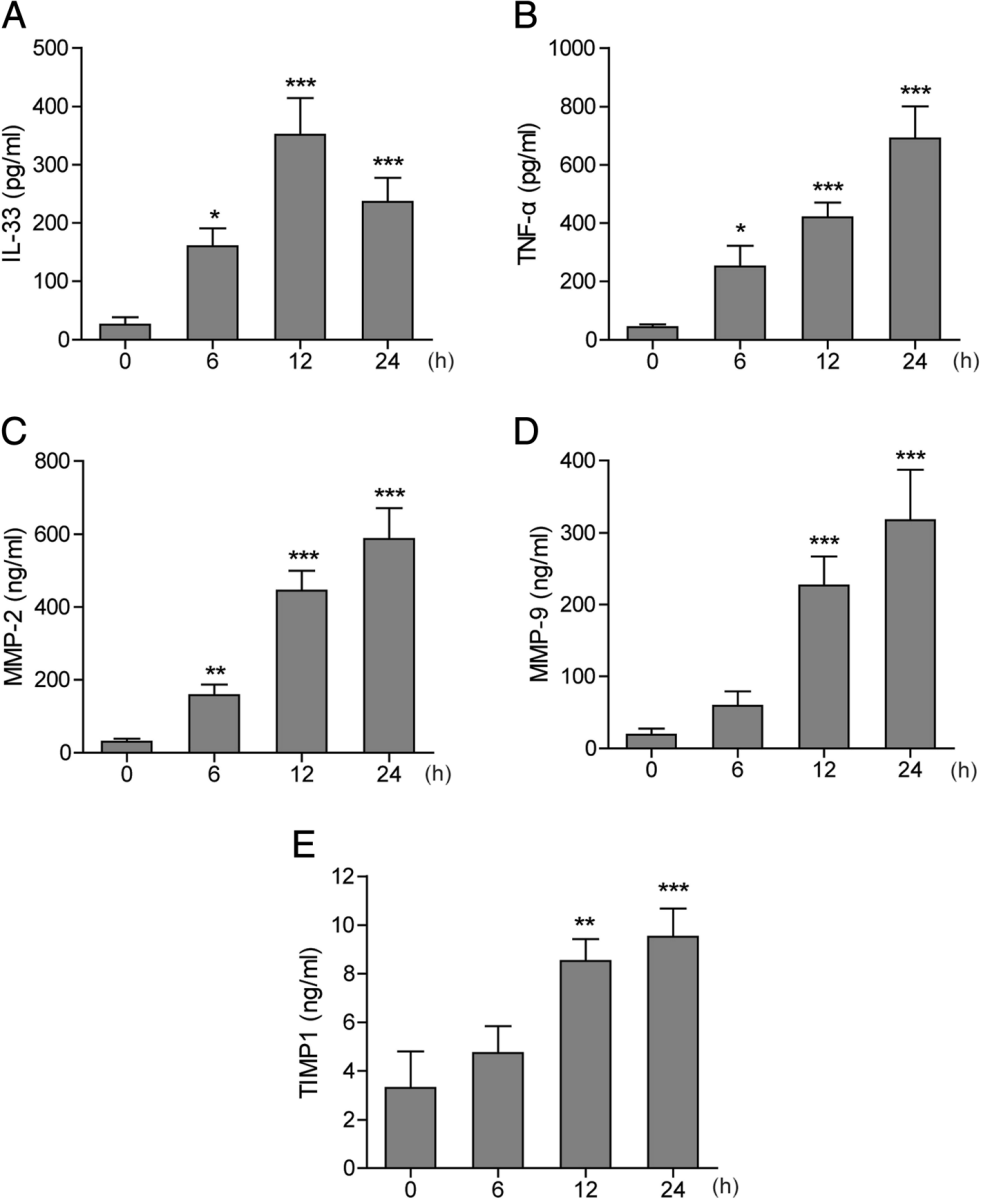
Levels of several inflammatory cytokines in culture medium of NR8383 cells after LPS stimulation. Levels of IL-33 (**a**), TNF-α (**b**), MMP2 (**c**), MMP9 (**d**), and TIMP1 (**e**) in the medium at indicated time points after LPS treatment (1 μg/mL) in NR8383 cells. Data are shown as means±SD (three independent repeats). One-way ANOVA was used in the statistical analyses. * *p* < 0.05, ** *p* < 0.01, *** *p* < 0.001 vs. zero time point

Considering the critical role of AMs in regulating pro-inflammatory events during ALI, we examined the effect of LPS on the secretion of IL-33, TNF-α, MMP2, and MMP9 in the AM cell line NR8383. Levels of TNF-α, MMP2, and MMP9 in culture medium increased in a time-dependent manner after LPS treatment, while the IL-33 level peaked at 12 h after LPS treatment (Fig. 3a-d). Although TIMP1 acts as a natural MMP inhibitor, the secretion of TIMP1 was also promoted by LPS (Fig. 3e). These results suggest that the levels of IL-33, TNF-α, MMP2, MMP9, and TIMP1 in BALF from the LPS-induced ALI rats may be raised by the alveolar macrophages which were activated by LPS.

### IL-33 increased expression of MMP2 and MMP9 via STAT3 signaling in AM cell line NR8383

**Fig. 4:**
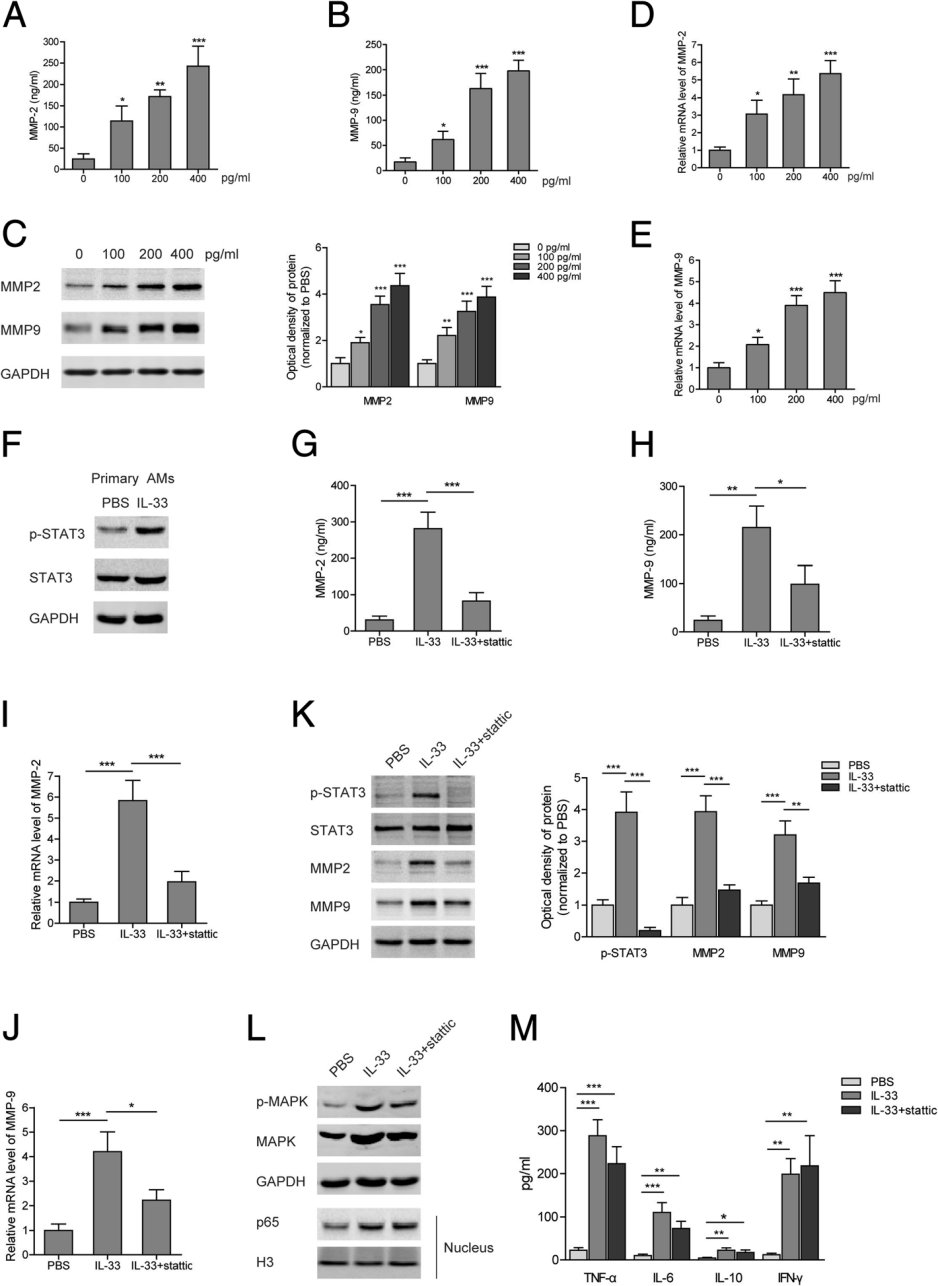
IL-33 increased expression of MMP2 and MMP9 via STAT3 signaling in NR8383 cells. **(a** and **b)** Levels of MMP2 (**a**) and MMP9 (**b**) in the medium at 24 h after IL-33 treatment with indicated concentrations in NR8383 cells. **(c**-**e)** Protein (**c**) and mRNA (**d** and **e**) levels of MMP2 and MMP9 at 24 h after IL-33 treatment with indicated concentrations in NR8383 cells. **(f)** Protein levels of p-STAT3 and STAT3 at 24 h after IL-33 treatment (400 pg/mL) in primary AMs. **(g** and **h)** Levels of MMP2 (**g**) and MMP9 (**h**) in the medium at 24 h after IL-33 (400 pg/mL) or stattic treatments in NR8383 cells. **(i**-**k)** Protein (**k**) and mRNA (**i** and **j**) levels of MMP2 and MMP9 at 24 h after IL-33 (400 pg/mL) or stattic treatments in NR8383 cells. **(l)** Protein levels of p-MAPK, MAPK, and p65 (within nucleus) at 24 h after IL-33 (400 pg/mL) or stattic treatments in NR8383 cells. **(m)** Levels of TNF-α, IL-6, IL-10, and IFN-γ in the medium at 24 h after IL-33 (400 pg/mL) or stattic treatments in NR8383 cells. Data are shown as means±SD (three independent repeats). The relative mRNA or protein level was normalized to 0 pg/ml (**c**-**e**) or PBS group (**i**-**k**). One-way ANOVA was used in the statistical analyses. * *p* < 0.05, ** *p* < 0.01, *** *p* < 0.001

**Fig. 5:**
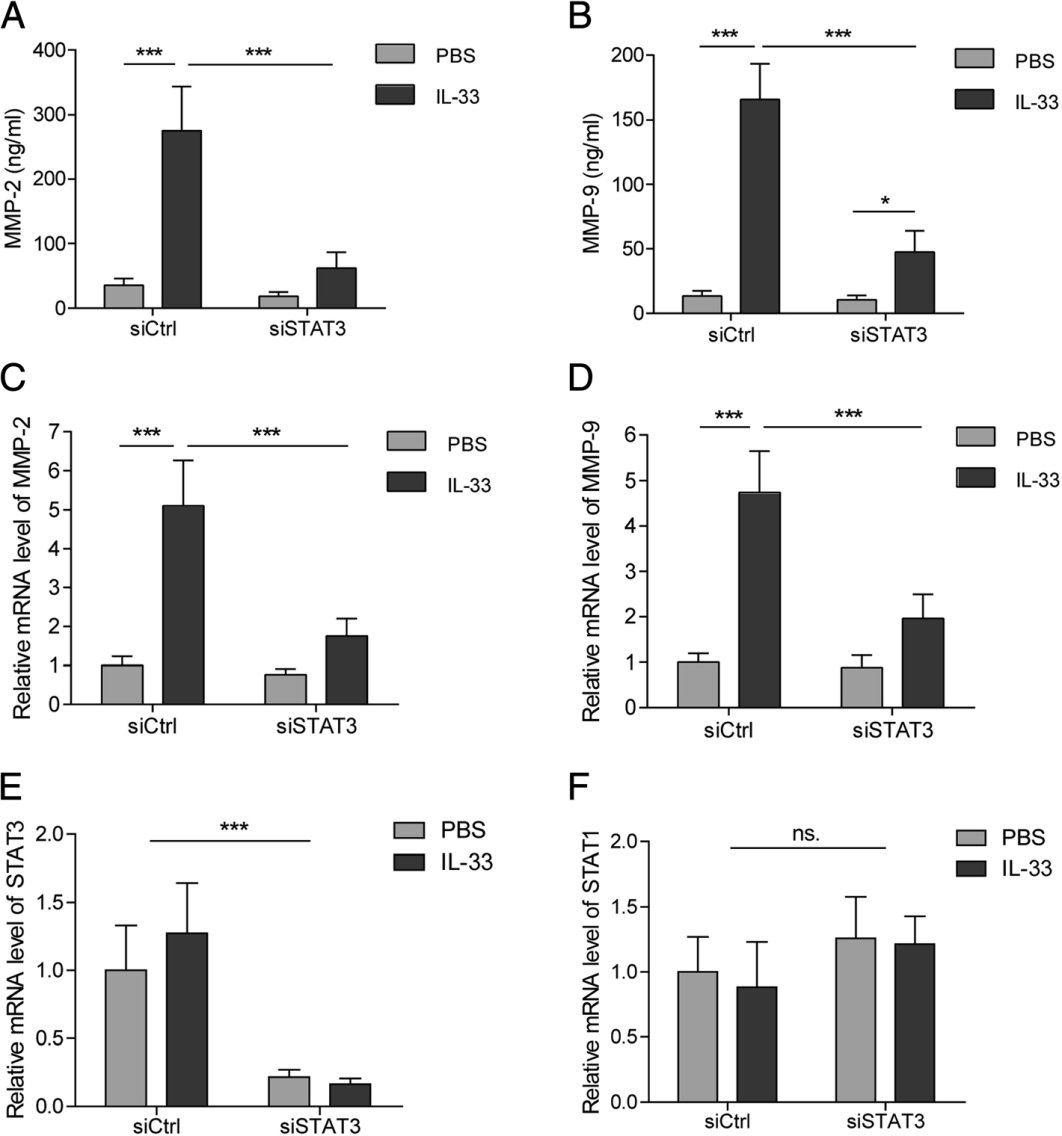
Effect of STAT3 knockdown on IL-33-induced expression of MMP2 and MMP9 in NR8383 cells. (**a** and **b)** Levels of MMP2 (**a**) and MMP9 (**b**) in the medium at 24 h after IL-33 treatment in NR8383 cells with or without knockdown of STAT3. **(c**-**f)** mRNA levels of MMP2 (**c**), MMP9 (**d**), STAT3 (**e**), and STAT1 (**f**) at 24 h after IL-33 treatment in NR8383 cells with or without knockdown of STAT3. Data are shown as means±SD (three independent repeats). The relative mRNA level was normalized to the siCtrl/PBS group. Two-way ANOVA was used in the statistical analyses. * *p* < 0.05, *** *p* < 0.001

To determine whether the secretion of MMP2 and MMP9 is induced by IL-33, NR8383 cells were stimulated with recombinant IL-33 protein. It was found that the concentrations of MMP2 and MMP9 in culture medium were increased in an IL-33-dose-dependent manner (Fig. 4a and b). Consistently, the mRNA and protein levels of MMP2 and MMP9 were upregulated in NR8383 cells treated with IL-33 (Fig. 4c-e). As a powerful signal transducer, STAT3 is essential for the interleukin-mediated activation of macrophages. Here, we found that IL-33 induced the phosphorylated activation of STAT3 in primary AMs (Fig. 4f). Blocking the activation of STAT3 with the specific inhibitor stattic attenuated the IL-33-induced expression and secretion of MMP2 and MMP9 in NR8383 cells, with no effect on activation of the MAPK or NFκb pathway (Fig. 4g-l). Additionally, we investigated whether other inflammatory cytokines were also produced through IL-33/STAT3 signaling in AMs. The results showed that IL-33 induced the secretion of TNF-α, IL-6, IL-10, and IFN-γ in NR8383 cells, which was not significantly changed after stattic addition (Fig. 4m). Similarly to stattic treatment, knocking down STAT3 with siRNA significantly decreased the mRNA levels of MMP2 and MMP9 in NR8383 cells as well as their concentrations in culture medium (Fig. 5a-f). Therefore, these results demonstrate that IL-33 promotes expression of MMP2 and MMP9 in AMs through activating STAT3.

### Neutralizing IL-33 inhibited the LPS-induced MMP2/9 expression and lung injury

**Fig. 6:**
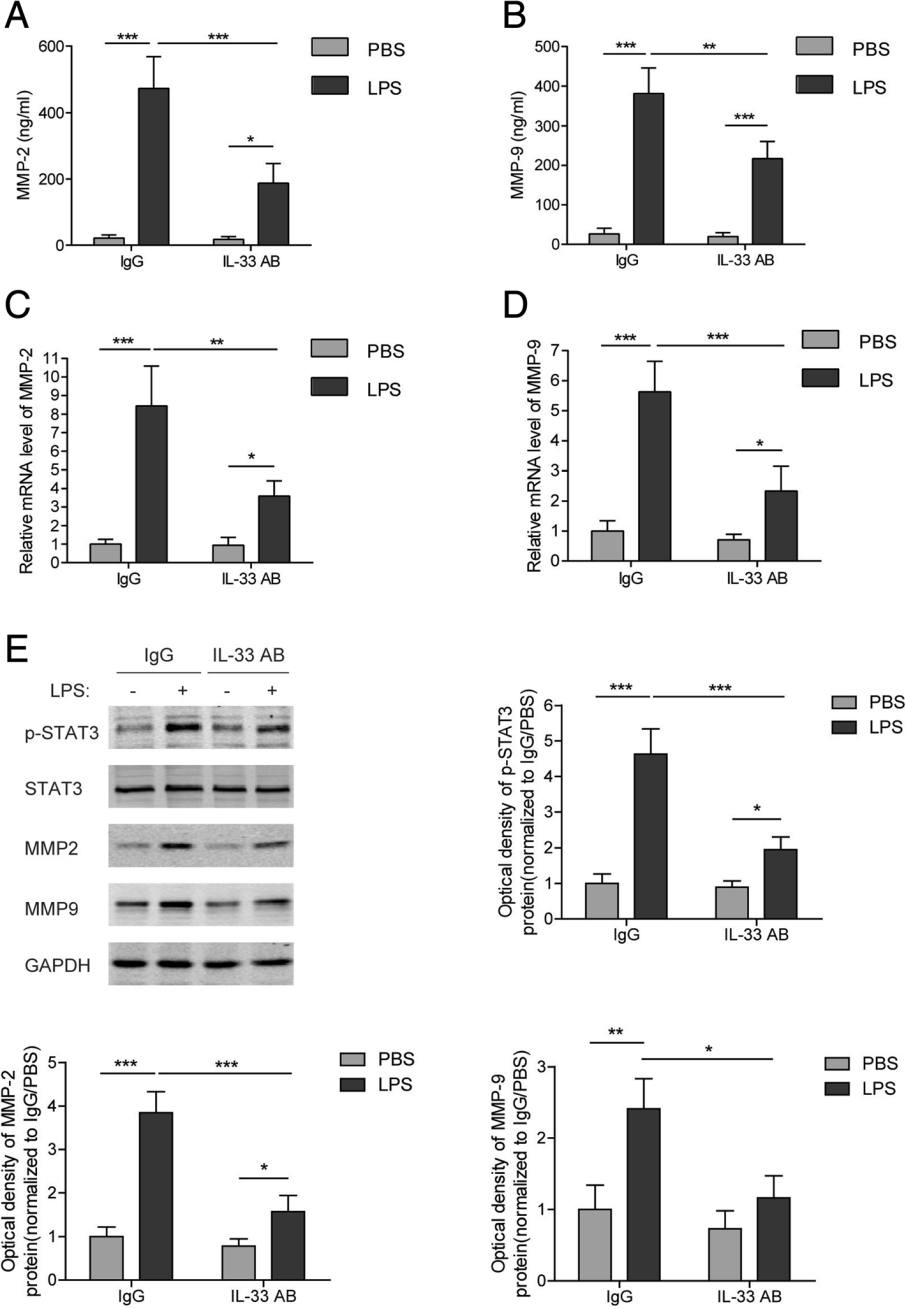
Effect of IL-33 neutralization on LPS-induced expression of MMP2 and MMP9 in NR8383 cells. **(a** and **b)** Levels of MMP2 (**a**) and MMP9 (**b**) in the medium at 24 h after LPS treatment with or without adding IL-33 antibodies to NR8383 cells. **(c**-**e)** mRNA (**c** and **d**) and protein (**e**) levels of MMP2 and MMP9 at 24 h after LPS treatment with or without adding IL-33 antibodies to NR8383 cells. Data are shown as means±SD (three independent repeats). The relative mRNA or protein level was normalized to the IgG/PBS group. Two-way ANOVA was used in the statistical analyses. * *p* < 0.05, ** *p* < 0.01, *** *p* < 0.001

**Fig. 7:**
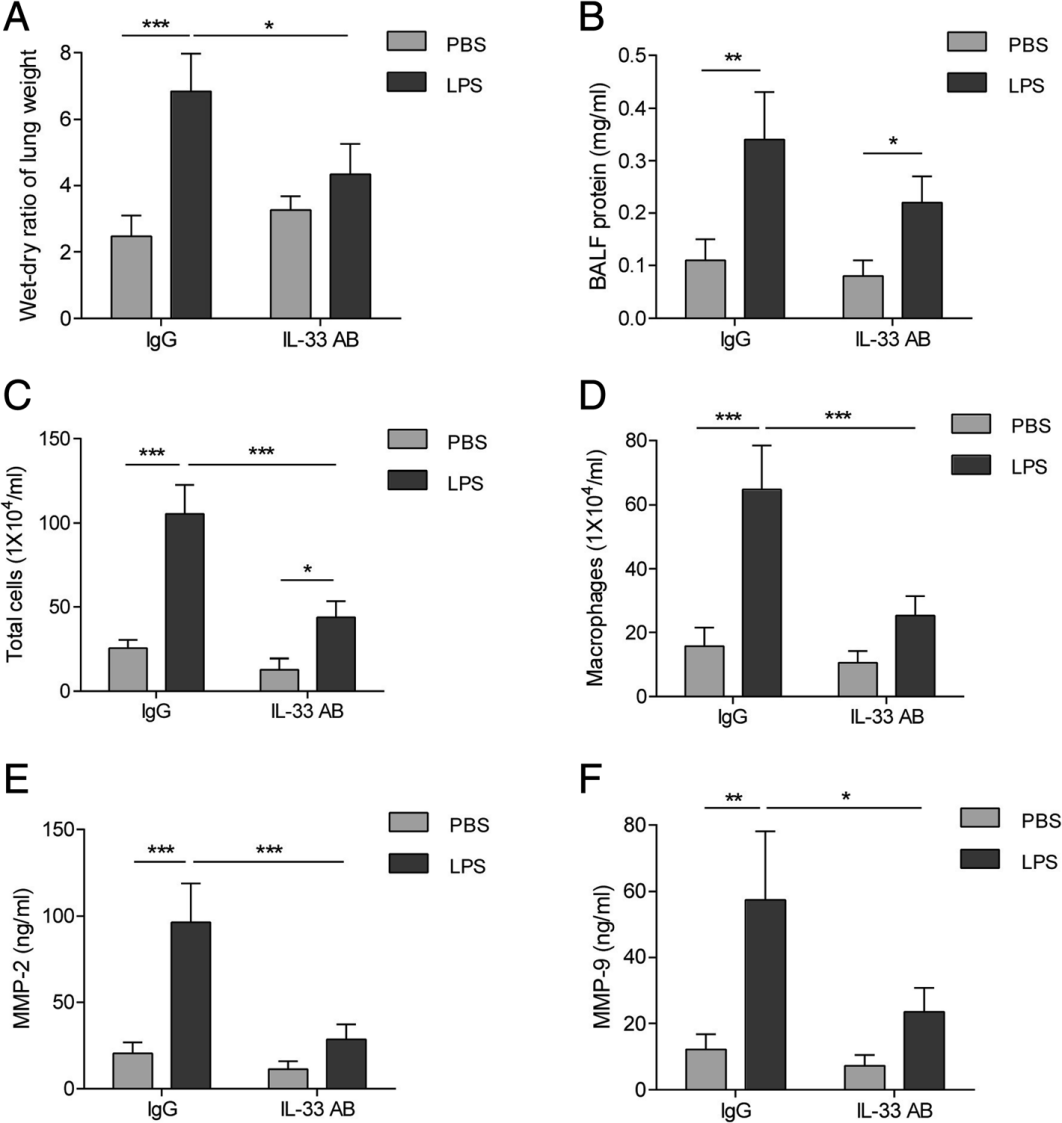
Effect of IL-33 neutralization on lung impairments and production of MMP2/9 in LPS-induced ALI rats. **(a**-**d)** Lung wet/dry ratio (**a**), protein contents in BALF (**b**), total cells in BALF (**c**) and macrophages in BALF (**d**) in LPS-induced ALI rats treated with or without IL-33 antibodies. **(e** and **f)** Concentrations of MMP2 (**e**) and MMP9 (**f**) in BALF of LPS-induced ALI rats treated with or without IL-33 antibodies. Data are shown as means±SD (*N* = 9 per group). Two-way ANOVA was used in the statistical analyses. * *p* < 0.05, ** *p* < 0.01, *** *p* < 0.001

To verify the role of IL-33 in LPS-induced ALI, IL-33 specific antibodies were administered into culture medium to neutralize the secreted IL-33 when AMs were being treated with LPS. It showed that IL-33 antibodies significantly lowered the mRNA and protein levels of MMP2 and MMP9 in LPS-treated NR8383 cells, as well as their medium concentrations (Fig. 6a-e). To further evaluate the protective effect of IL-33 antibody in the LPS-induced ALI model, the rats received an intratracheal instillation of IL-33 antibody after LPS administration to neutralize the secreted IL-33. The results showed that treatment with anti-IL-33 antibodies obviously curbed the edema development and inflammatory cell infiltration in the lung tissues from LPS-induced ALI rats (Fig. 7a-d). Moreover, the concentrations of MMP2 and MMP9 in BALF from the LPS-treated rats were substantially decreased after the addition of IL-33 antibodies (Fig. 7e and f). Taken together, IL-33 displays a central role in LPS-induced lung inflammation/injury and MMP2/9 secretion.

## Discussion

In the present study, an in vivo LPS-induced ALI rat model and an in vitro model using AM NR8383 cells were established to investigate the link between IL-33 and ALI. BALF from the in vivo model showed an increased IL-33 level, which is consistent with the observation in LPS-induced ALI mice [14]. The expression of IL-33 was boosted by LPS in AMs, suggesting the ability of AMs to produce IL-33 during LPS-induced ALI. AMs, once activated by toll-like receptors (TLRs), serve as the first line of defense against invaders into the lung [15]. Wang et al. reported that the NF-κB pathway aberrantly activated in BALF-derived macrophages aroused ARDS in rats [16]. Therefore, we supposed that the LPS-induced IL-33 secretion requires activation of the TLR4/NF-κB pathway since NF-κB is the central mediator of inflammation. Several studies have demonstrated that IL-33 directly up-regulated expression of the LPS receptor TLR4 in macrophages, and this up-regulation in turn exaggerated the activation of NF-κB and increased the production of pro-inflammatory cytokines [17, 18]. Furthermore, Fu et al. found that the up-regulation of IL-33 in LPS-induced ALI mice also involved HMGB1, a leading factor for the cascade amplification of inflammation [19].

Except for IL-33, LPS stimulation immediately activated MMP2 and MMP9 production in BALF from ALI rats, which is also validated by the results of in vitro studies using NR8383 cells. However, the level of IL-33 secreted by NR8383 cells peaked at 12 h after LPS treatment, while levels of MMP2 and MMP9 continued to increase, more evidently after 12 h. As the time-course study revealed that relatively early expression of IL-33 was followed by increased production of MMP2 and MMP9 after LPS challenge in AMs, we hypothesize that autocrine or paracrine IL-33 release after LPS stimulation further activates the downstream inflammatory pathways which up-regulate the expression of MMP2 and MMP9. Neutralizing the secreted IL-33 after LPS treatment not only decreased the production of MMP2 and MMP9 but also protected the rats from LPS-induced ALI, indicating that IL-33 may function as an inflammatory mediator in ALI development.

A few studies have been conducted to investigate the mechanisms behind IL-33-mediated expression of MMP2 and MMP9 in macrophages. Ariyoshi et al. found that the transcription of MMP9 was induced by AP-1 activation, which is dependent on ERK1/2/CREB and NF-κB cascades in IL-33-stimulated macrophages [20]. However, in the present study, STAT3 was identified as a required mediator in IL-33-induced expression of MMP2 and MMP9. Although STAT3 directly activates the transcription of MMP2 and MMP9 in cancer cells [21, 22], the regulatory mechanisms in alveolar macrophages should be further validated.

In conclusion, IL-33 production increases in LPS-induced ALI rats and LPS-treated alveolar macrophages. Then, the secreted IL-33 induces the expression of MMP2 and MMP9 in a STAT3-dependent manner. Disrupting the IL-33/STAT3/MMP2/9 pathway by neutralizing IL-33 relieves the pulmonary inflammation and injury in LPS-induced ALI rats, suggesting that IL-33 could be a potential therapeutic target for ALI.

## Abbreviations

ALI: Acute lung injury
AMs: Alveolar macrophages; TLRs: toll-like receptors
ARDS: Acute respiratory distress syndrome
BALF: Bronchoalveolar lavage fluid
IL: Interleukin
LPS: Lipopolysaccharide
MMPs: Matrix metalloproteinases
